# Antibacterial Electroconductive Composite Coating of Cotton Fabric

**DOI:** 10.3390/ma15031072

**Published:** 2022-01-29

**Authors:** Tomasz Makowski, Mariia Svyntkivska, Ewa Piorkowska, Urszula Mizerska, Witold Fortuniak, Dorota Kowalczyk, Stefan Brzezinski, Dorota Kregiel

**Affiliations:** 1 Centre of Molecular and Macromolecular Studies Polish Academy of Sciences, Sienkiewicza 112, 90-363 Lodz, Poland; mariiasv@cbmm.lodz.pl (M.S.); mizerska@cbmm.lodz.pl (U.M.); wfortuni@cbmm.lodz.pl (W.F.); 2 Lukasiewicz Research Network, Textile Research Institute, Brzezinska 5/15, 92-103 Lodz, Poland; dorota.kowalczyk@iw.lukasiewicz.gov.pl (D.K.); awbrzezi@toya.net.pl (S.B.); 3 Department of Environmental Biotechnology, Faculty of Biotechnology and Food Sciences, Lodz University of Technology, Wolczanska 171/173, 90-924 Lodz, Poland; dorota.kregiel@p.lodz.pl

**Keywords:** electroconductive cotton, graphene oxide, electrochemical functionalization, antibacterial activity

## Abstract

Graphene oxide (GO) was deposited on a cotton fabric and then thermally reduced to reduced graphene oxide (rGO) with the assistance of L-ascorbic acid. The GO reduction imparted electrical conductivity to the fabric and allowed for electrochemical deposition of Ag° particles using cyclic voltammetry. Only the Ag°/rGO composite coating imparted antibacterial properties to the fabric against *Escherichia coli* and *Staphylococcus aureus*. Ag°/rGO-modified fibers were free of bacterial film, and bacterial growth inhibition zones around the material specimens were found. Moreover, Ag°/rGO-modified fabric became superhydrophobic with WCA of 161°.

## 1. Introduction

Electroconductive textiles draw increasing attention because of their numerous potential applications including sensors, solar cells, and supercapacitors [1,2,3,4]. Electroconductivity can be achieved through coating of textiles with carbon nanoparticles, such as carbon nanotubes or graphene materials (GM), including graphene or reduced graphene oxide (rGO), which form conductive networks on fiber surfaces [3,5,6,7,8]. Despite the outstanding properties of graphene [3], its use for modification of textiles is limited by difficulty of obtaining stable aqueous dispersions applicable to hydrophilic natural fibers. In turn, functional groups of graphene oxide (GO) facilitate its dispersion in water but adversely influence its electrical conductivity, hence GO has to be reduced chemically or thermally to rGO. Chemical reduction of GO often requires elevated temperature, 80–100 °C, and a relatively long time, even several hours or days, whereas thermal reduction usually demands even higher temperature and oxygen-free conditions [9,10,11].

In our previous studies, cotton fabric was made electroconductive by coating with GO and its reduction at 220 °C, assisted with L-ascorbic acid or commercial antioxidants used in the plastic industry, resulting in hydrophilicity or hydrophobicity of the fabric, respectively [12,13]. It is worth mentioning that the decomposition of cotton occurs above 240–250 °C [14].

Presently, there is a growing demand for smart and intelligent garments, including cotton cloth, that are electroconductive and able to store energy or communicate. In such applications, antimicrobial activity can eliminate microbial colonization and reduce the possibility of infection. GM particles exhibit antibacterial activity, dependent on their dispersibility, adsorption ability, number of corners and sharp edges [15], hence strongly influenced by the lateral size, shape, number of layers, surface modification, agglomeration, and dispersion. Three main mechanisms, recently postulated, include the action of sharp edges, oxidative stress, and wrapping or trapping of bacteria due to the flexible thin-film structure of GM. The action of the sharp edges, also called nanoknives, nanoblades, or cutters, is believed to be crucial. The edges, acting as cutters, can mechanically disrupt the cellular membranes and cause their integrity loss, e.g., [16,17,18], although it requires the direct contact of the bacteria with GM sheets. Recently, to obtain an antibacterial material, Han et al. [19] functionalized GO with hydrophilic polymers and used it as a vector for silver (Ag) nanoparticles and sulfadiazine. Deposition of Ag particles on carbon nanotube-coated fibrous materials, including cotton fabric, made them antibacterial [6,20,21], as such particles act against bacteria and fungi, and are also antiviral [22,23]. Metal-based nanoparticles are effective against a wide variety of microorganisms as their bonds to biomolecules are non-specific [23,24,25,26,27]. 

However, only a few articles described modification of cotton fabric through chemical reduction of GO and deposition of Ag [28,29,30]. It improved hydrophobicity and electrical conductivity, but antibacterial activity was not reported. Recently, GO-coated linen and cotton fabrics were made antibacterial through simultaneous chemical reduction of GO and deposition of Ag [21]. The antibacterial properties of the fabric modified solely with rGO were not examined.

In the present study, to make it electroconductive, cotton fabric was GO-coated and then GO was reduced thermally to rGO. Ag° particles were deposited electrochemically on rGO-coated fabric with cyclic voltammetry, which allowed to monitor the redox reactions. The composite Ag°/rGO coating imparted antibacterial activity to the fabric against the tested bacterial strains, *Escherichia coli* and *Staphylococcus aureus,* and also superhydrophobicity.

## 2. Experimental Section

For further modification, white plain weave cotton fabric with 205 threads/10 cm and 295 threads/10 cm in the warp and weft direction, respectively, was used. It was 0.36 mm thick, with a surface density of 145 g/m^2^. The fabric was cleaned by extraction [5]. 

To coat the fabric, 0.6 wt.% aqueous dispersion of GO was used from Graphene Laboratories, Inc. (Calverton, NY, USA) (single layer > 80%, flake size of 0.5–5 μm). 

Thermal reduction of GO on the fabric was assisted with L-ascorbic acid (C_6_H_8_O_6_), (POCH, Poland, 98%). Silver nitrate (AgNO_3_) and trisodium citrate (Na_3_C_6_H_5_O_7_) (Avantor, Poland, ≥99%) were used for electrochemical deposition of Ag° particles.

The aqueous dispersion of GO was diluted with water to decrease GO concentration to 0.1 wt.% and was homogenized for 20–30 min at room temperature (RT) using ultrasonic homogenizer HielscherUP 200S (Teltow, Germany). The dispersion was deposited on the fibers by the padding method using a laboratory double-roll padding machine (BENZ, Switzerland) with horizontally set squeezing rollers. The details of the padding procedure are described elsewhere [12]. 

Before the reduction, GO-coated fabric was four times dipped in 0.1 wt.% aqueous solution of L-ascorbic acid and next dried at RT. The reduction was carried out in a Mettler Toledo Hot Stage FP82 (Greifensee, Switzerland) equipped with an FP90 temperature controller. As previously described [12], GO-coated specimens, placed between microscope glasses, were heated from RT to 220 °C at 10 °C/min, annealed for 1 min, and cooled down to RT at 10 °C/min. In this way, rGO-coated fabric was obtained. The influence of such thermal treatment on mechanical properties of the fabric was examined by us previously [12,13]. 

Silver particles (Ag°) were deposited on rGO-coated fabric with cyclic voltammetry using BioLogic SP-150 (Bio-Logic Science Instruments SAS, Claix, France) potentiostat/galvanostat, during five cycles in a mixture of aqueous solutions of sodium citrate and silver nitrate, with a platinum (Pt°) electrode and a silver chloride electrode (Ag|AgCl), as the counter and reference electrodes, respectively. The process was controlled and monitored with EC-Lab Electrochemistry v11.32 software. The reduction of Ag^+^ ions occurred in the voltage range of ±1.0 V vs. Ag|AgCl [20]. To remove Ag^+^ ions and salts formed during the reaction, after the process the Ag°/rGO-coated fabric was rinsed in distilled water three times and dried at RT. 

The Ag content was determined by thermogravimetric analysis (TGA), using TA Instruments TGA 5500 (New Castle, DE, USA) during heating at 20 °C/min in air.

Surface electrical resistivity (R) of the fabric was measured using the four-wire method with Keithley 2400C SourceMeter (Tektronix, Beaverton, OR, USA). Copper electrodes were attached to the surface with silver-containing paste Dotite D-550 (Fujikura Kasei, Tokyo, Japan), 1 cm apart. The measurements were repeated at least three times to obtain average values of R.

The fabric samples were vacuum sputtered with a 10 nm gold layer using Quorum EMS150R ES (Quorum Technologies, Laughton, UK), and then analyzed using a scanning electron microscope (SEM), and also SEM with energy dispersive spectroscopy (EDS), JEOL 6010LA (Tokyo, Japan), at an accelerating voltage of 10 kV. 

To determine water wettability, 5 µL distilled water droplets were placed on the examined surfaces and water contact angles (WCA) were measured at 25 °C by means of a RameHart NRL Goniometer 100-00-230 (Succasunna, NJ, USA) coupled to a camera and optical system, using Drop Analysis program. The measurements were repeated five times to obtain average WCA values.

Antibacterial properties of the fabric samples against Gram-positive *Staphylococcus aureus* ATCC 6538 and Gram-negative *Escherichia coli* ATCC 8739 were tested using Antibacterial Activity Assessment of Textile Materials: Agar Diffusion Test (ISO20645:2004) [31]. 15 mL of nutritive agar medium, Tripticase Soy Agar from Merck (Darmstadt, Germany), were poured on Petri dishes. Inoculums of microbial cultures of 1–5 × 10^8^ CFU/mL (0.2 mL) were then poured on the agar media. Disk specimens, with 5 mm diameter, were placed on the agar surface. After 48 h incubation at 37 °C, the contact zones under the tested specimens were analyzed visually, and then the inhibition zones, if present, were measured. The fibers were also analyzed by SEM, to evaluate the bacterial colonization. 

## 3. Results and Discussion

The reduction of GO deposited on the fabric made the fabric electrically conductive. When the temperature of the hot stage approached 220 °C, R diminished significantly but at 220 °C it leveled off within 1 min at the value of 0.1 MΩ/sq. During cooling and further storage at RT, R increased but finally stabilized after 24 h. Such behavior was observed by us previously [12,13,32] due to partial reversibility of the reduction. Nevertheless, R of 5.4 MΩ/sq was achieved, showing the presence of conducting 3D-network of rGO on fiber surfaces. 

**Figure 1:**
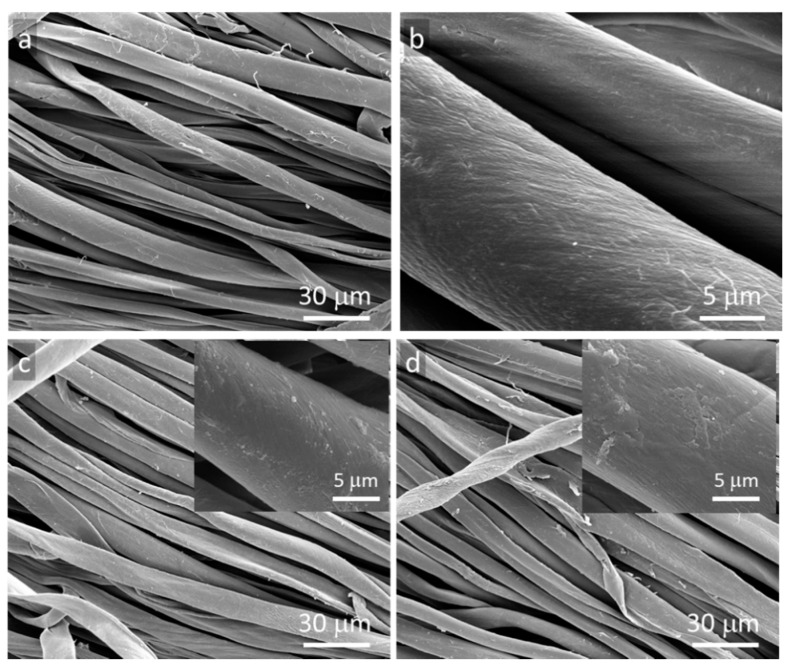
SEM micrographs of cotton fabric: (**a**,**b**) neat, (**c**) GO-coated, (**d**) rGO-coated.

SEM micrographs of GO and rGO-coated fibers are shown in Figure 1. Small thickness and very good adhesion to fiber surfaces make the particles hardly visible on the fibers [13]. However, the areas without typical morphology of cotton fibers are discernible, shown in the insets, which are suggestive of GO or rGO thin layers.

**Figure 2:**
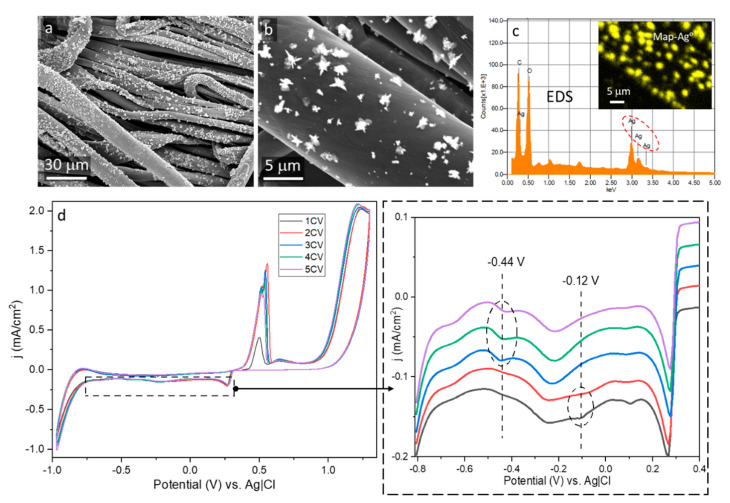
Ag°/rGO-coated cotton fabric: (**a**,**b**) SEM micrographs, (**c**) EDS Ag mapping and spectrum, (**d**) cyclic voltammetry profiles.

Reduction of Ag^+^ ions occurred on the rGO-coated surfaces, combined with nucleation and growth of Ag° particles, which are visible in SEM micrographs in Figure 2a,b. EDS analysis confirmed the presence of Ag° particles on rGO-coated surface, as shown in Figure 2c. Reaction of silver nitrate with sodium citrate resulted in the formation of silver citrate complex stabilized by carboxyl groups of citric acid, which prevented aggregation of Ag° particles and enabled development of particles immobilized on the surface [33]. The most effective complexing occurs when [citrate]/[Ag^+^] >> 1 [34]. Others [35] studied GO electrochemistry and found that the reduction reactions occurred on edges of GO plates. Thus, we postulate that the reduction of the complex and the formation of Ag° particles, through nucleation and growth, could occur on edges or defects of rGO plates.

**Scheme 1:**
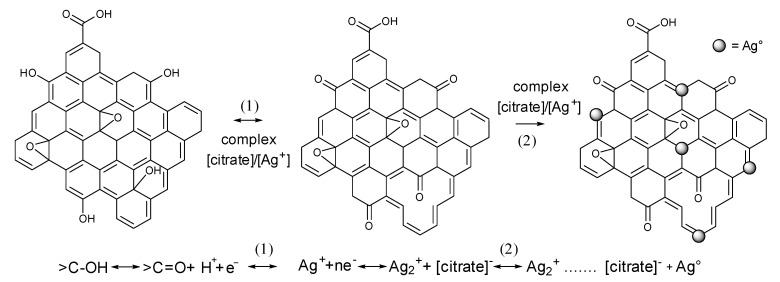
Proposed mechanism of Ag° formation on rGO deposited on cotton fiber surfaces.

The voltammetry profile during the first and the second cycle, shown in Figure 2d, exhibits one redox weak peak at −0.12 V attributed by others to reduction of hydroxyl groups [36]. Most probably, it was related to residual hydroxyl groups of rGO, remaining due to its incomplete thermal reduction. Further reactions postulated in [36] were not observed due to a small number of other functional groups on rGO surface and competitive reactions involving Ag^+^ ions. During the next three cycles, a peak at −0.44 V evidenced the reduction of Ag^+^ ions. The proposed mechanism of the formation of the Ag° particles is shown in Scheme 1. 

Based on the results of TGA experiments, by taking into account the residue weight at 900 °C of Ag°/rGO-modified fabric and rGO-modified fabric, the Ag content of 0.4 wt.% was determined. The successful deposition of Ag° particles confirmed the formation of the electroconductive rGO network on fiber surfaces and indicated that rGO-coated fabrics can serve as electrodes in cyclic voltammetry. R of the fabric did not change after the electrochemical modification and was equal to 5.35 MΩ/sq because Ag° was deposited on the rGO as separate particles, which did not form any continuous network. However, the Ag°/rGO-modified fabric became superhydrophobic with WCA of 161°, due to the presence of hydrophobic Ag° particles on the surface, causing the lotus effect.

**Figure 3:**
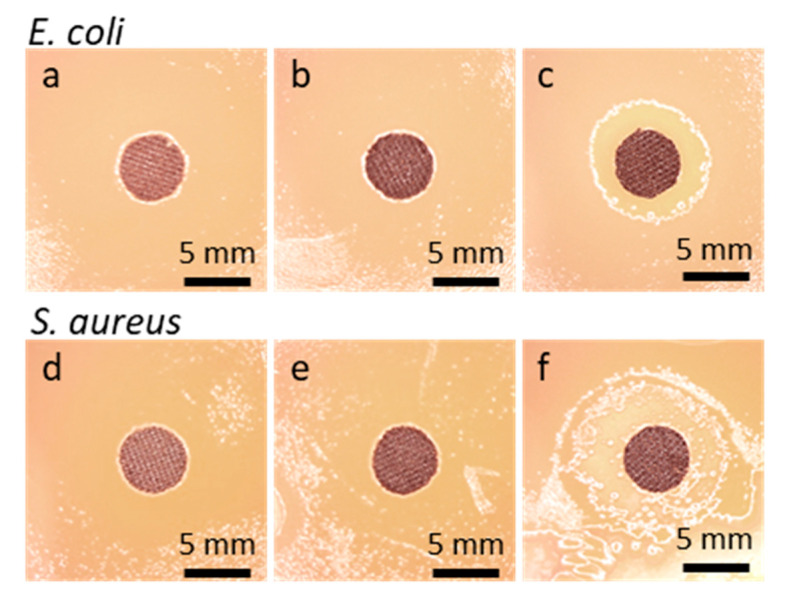
Disk specimens of cotton fabric on bacteria inoculated agar after 48 h incubation: *E. coli* (**a**) GO-coated, (**b**) rGO-coated, (**c**) Ag°/rGO-coated; *S. aureus*, (**d**) GO-coated, (**e**) rGO-coated, (**f**) Ag°/rGO-coated.

As seen in Figure 3, no bacterial growth inhibition zone was found around GO-coated or rGO-coated fabric specimens. The growth inhibition zones of about 9 mm and 12 mm for Gram-positive *Staphylococcus aureus* and Gram-negative *Escherichia coli*, respectively, were only observed around the specimens of Ag°/rGO-coated fabric.

Ag° particles exhibit non-specific bacterial toxicity that impedes the bacteria resistance development and also broadens the spectrum of antibacterial activity [37]. The main activity mechanisms of metal nanoparticles or ions include attraction to bacterial cell walls, disruption of the cell walls increasing their permeability, and disruption of cell functions [23,24,25,37,38]. 

**Figure 4:**
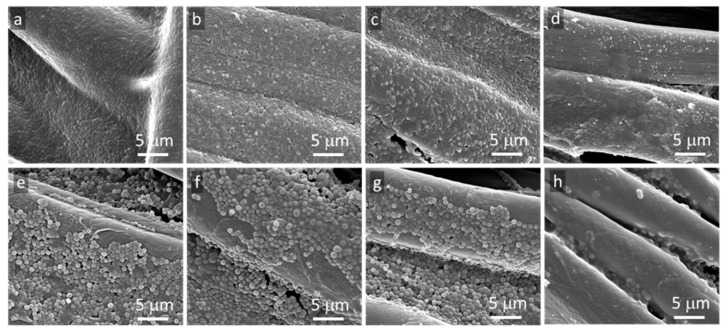
SEM micrographs of bottom surfaces of cotton fabric specimens removed from agar after 48 h incubation: *E. coli* (**a**) neat fabric, (**b**) GO-coated, (**c**) rGO-coated, (**d**) Ag°/rGO-coated, *S.aureus* (**e**) neat fabric, (**f**) GO-coated, (**g**) rGO-coated, (**h**) Ag°/rGO-coated.

The absence of bacterial growth inhibition zones around specimens does not exclude their antibacterial activity in direct contact with microorganisms. Therefore, SEM was used to analyze the bottom surfaces of the tested fabric specimens. SEM micrographs of bottom surfaces of cotton fabric specimens in Figure 4, modified and unmodified, show that not only the neat fibers but also GO and rGO-coated fibers were inhabited by both tested bacterial strains.

According to the literature, there are three basic mechanisms of antibacterial activity of GO. The first one, the most commonly observed, is the effect of sharp edges, known as nanoblade effect. The nanoblades cut cell membranes causing their damage leading to the death of bacteria [16,17,18]. The second one is oxidative stress caused by reactive oxygen species, leading to bacteria DNA damage and mitochondrial dysfunction [39]. The third mechanism reported, observed rather in solutions than in coatings, is wrapping or trapping of bacteria by GO flakes, which causes their isolation from the environment [40]. The main mechanisms of antibacterial activity of rGO are related to the nanoblade effect and oxidative stress [15]. In the current study, GO or rGO flakes adhering to cotton fibers and lying flat on them did not expose sharp edges, and no wrapping or trapping of bacteria was observed by SEM. Therefore, their antibacterial activity was greatly limited. To achieve electroconductivity, a sufficient amount of GO particles had to be deposited on the fiber surfaces to form a 3D-newtork, which was then made electroconductive by GO reduction. However, the formation of 3D-network did not require full coverage of fibers with GO or rGO platelets. The uncovered areas, without coating, were prone to bacteria colonization. In turn, the activity of Ag° particles is well known and includes the release of Ag^+^ through oxidative dissolution, in which atmospheric O_2_ dissolved in water plays a role of oxidizer [38]. Due to the diffusion of thus formed ions, the antibacterial activity of Ag° modified surface does not require a close contact with microorganisms, which is reflected in the bacterial growth inhibition zones around the specimens in Figure 3.

## 4. Conclusions

The cotton fabric was coated with GO dispersion using the padding method, and then GO was reduced thermally to rGO with the assistance of L-ascorbic acid. Then, on the electroconductive rGO network, Ag° particles were electrochemically deposited with cyclic voltammetry, which enabled monitoring the redox reactions. Antibacterial activity of the modified cotton fabric against Gram-positive *Staphylococcus aureus* and Gram-negative *Escherichia coli* bacteria was tested using a disk diffusion method. Neither before nor after the GO reduction the coated fabric exhibited antibacterial activity. This was reflected in the colonization of the fibers by the bacteria, as shown by SEM, and the absence of the bacterial growth inhibition zones around the fabric specimens. Only the Ag^o^/rGO coating imparted antibacterial properties to the fabric reflected in bacterial inhibition growth zones around the tested specimens and the absence of bacterial films on the fibers. Moreover, Ag°/rGO-modified fabric became superhydrophobic with WCA of 161°. The obtained results on composite Ag°/rGO coating of the fabric seem to be promising for obtaining novel antibacterial materials.

## Data Availability

The data are available on request from the corresponding author.
